# Signaling Pathway Dissection After Progesterone Receptor Enhancement in an Immortalized Pre-Cancer Fallopian Tube Epithelial Cell Line

**DOI:** 10.3390/ijms27094031

**Published:** 2026-04-30

**Authors:** Yu-Hsun Chang, Kun-Chi Wu, Dah-Ching Ding

**Affiliations:** 1 Department of Pediatrics, Hualien Tzu Chi Hospital, Buddhist Tzu Chi Medical Foundation, Tzu Chi University, Hualien 970, Taiwan; cyh0515@gmail.com; 2 Department of Orthopedics, Hualien Tzu Chi Hospital, Buddhist Tzu Chi Medical Foundation, Tzu Chi University, Hualien 970, Taiwan; drwukunchi@yahoo.com.tw; 3 Department of Obstetrics and Gynecology, Hualien Tzu Chi Hospital, Buddhist Tzu Chi Medical Foundation, Tzu Chi University, Hualien 970, Taiwan; 4 Institute of Medical Sciences, Tzu Chi University, Hualien 970, Taiwan

**Keywords:** progesterone receptors, FE25, fallopian tube epithelial cells, p53, retinoblastoma

## Abstract

Ovarian cancer remains the most lethal gynecologic malignancy, with the majority of patients presenting at advanced stages and exhibiting poor long-term survival. High-grade serous carcinoma (HGSC), the predominant subtype, likely originates from fallopian tube epithelial cells (FTECs), whose biology is strongly influenced by hormonal signaling. Progesterone receptor (PR) expression, particularly of the PR-B isoform, is associated with improved prognosis in HGSC; however, the isoform-specific molecular mechanisms in precancerous FTECs remain unclear. This study investigated the distinct biological and transcriptomic effects of PR-A and PR-B in p53- and Rb-defective FE25 FTEC-derived cells. Stable overexpression of PR-A suppressed cell proliferation, enhanced apoptosis, and induced robust senescence, whereas PR-B promoted proliferation and activated JNK/c-Jun signaling. Upon progesterone (P4) treatment, both isoforms mediated cell-cycle arrest and apoptosis, with PR-A exhibiting stronger Sub-G1 induction. PR-A and PR-B differentially regulated cell-cycle inhibitors, senescence markers, and downstream pathways, including the PI3K–Akt and MAPK pathways, while RNA sequencing analyses revealed broader P4-induced transcriptomic changes in PR-B than in PR-A, involving immune, angiogenic, and proliferative programs. Collectively, these findings demonstrate that PR-A and PR-B exert distinct yet complementary regulatory roles in FTEC biology and progesterone responsiveness. The observed PR isoform-dependent effects in FE25 cells should be interpreted as context-specific mechanistic insights rather than direct predictors of clinical prognosis or treatment response.

## 1. Introduction

The incidence rate of ovarian cancer in the United States is approximately 11.4 cases per 100,000 women annually, with a lifetime risk of about 1.1% in the general population [1]. The 5-year survival rate for ovarian cancer is highly dependent on the stage at diagnosis. For localized (stage I) disease, the 5-year survival rate is approximately 92.5%, whereas for distant (stage III–IV) disease, it declines to 28.9% [2]. The majority of the patients (approximately 80%) present with advanced-stage disease, contributing to the relatively poor overall survival [3]. High-grade serous carcinoma (HGSC) is the most common histology in ovarian cancer, which may arise from the fallopian tube epithelial cells (FTECs) [4]. Hormonal influences may alter FTEC gene expression, providing new insights into their regenerative responses to hormonal signaling [5]. The progesterone receptor (PR) plays distinct roles in regulating FTEC behavior, and targeting PR could be a potential therapeutic strategy for ovarian cancer treatment [6].

PR expression serves as a favorable prognostic biomarker in ovarian cancer, particularly in HGSC and endometrioid subtypes [7,8]. Strong PR expression is independently associated with improved disease-specific and overall survival, with hazard ratios indicating a significant reduction in mortality risk for PR-positive tumors compared with PR-negative tumors [8,9]. This prognostic benefit is most pronounced in endometrioid carcinoma and HGSC and is less evident or absent in mucinous and clear cell subtypes [9,10,11]. Mechanistically, PR activation may induce apoptosis and reflect a less aggressive tumor phenotype, possibly indicating a functionally intact estrogen receptor pathway [12]. The ER–PR+ phenotype is associated with early-stage disease, higher differentiation, and long-term survival [13]. Among the PR isoforms, PR-B has been identified as an independent marker for improved survival [14].

PR exists in two forms, PR-A and PR-B, each encoded by the same gene but differing in their N-terminal structure and transcriptional activity [14]. PR-B is generally expressed at higher levels than PR-A in epithelial ovarian carcinoma, and its presence is associated with improved survival [15]. Multivariate analyses have shown that PR-B, but not PR-A, is an independent prognostic factor for patient survival, with higher PR-B expression correlating with better progression-free and overall survival, particularly in HGSC and endometrioid subtypes [15,16]. Mechanistically, PR-B may promote apoptosis and enhance sensitivity to platinum-based chemotherapy, further contributing to a favorable prognosis [15]. In contrast, PR-A, while present, does not consistently demonstrate independent prognostic value in ovarian cancer. The differential expression and function of PR-A and PR-B suggest that assessing PR isoforms, especially PR-B, may provide more precise prognostic information and inform individualized therapeutic strategies [17].

HGSC is characterized by near-universal TP53 dysfunction and frequent disruption of cell-cycle regulatory pathways, including RB signaling [18]. FE25 cells exhibit alterations in both p53 and RB pathways while retaining a non-fully malignant phenotype, modeling an early epithelial state prior to overt transformation [19]. As such, FE25 cells provide a useful platform to interrogate progesterone receptor isoform-dependent signaling in a molecular context that mirrors key HGSC-defining alterations [20]. Insights gained from this model may therefore inform how early PR isoform imbalance influences downstream pathways relevant to HGSC initiation and progression.

This study aimed to elucidate the distinct biological and transcriptomic effects of PR isoforms PR-A and PR-B on proliferation, cell-cycle regulation, apoptosis, and progesterone responsiveness in precancerous p53- and Rb-defective FTECs (FE25).

## 2. Results

### 2.1. Differential Effects of PR-A and PR-B Overexpression on Proliferation in FE25 Cells

**Figure 1:**
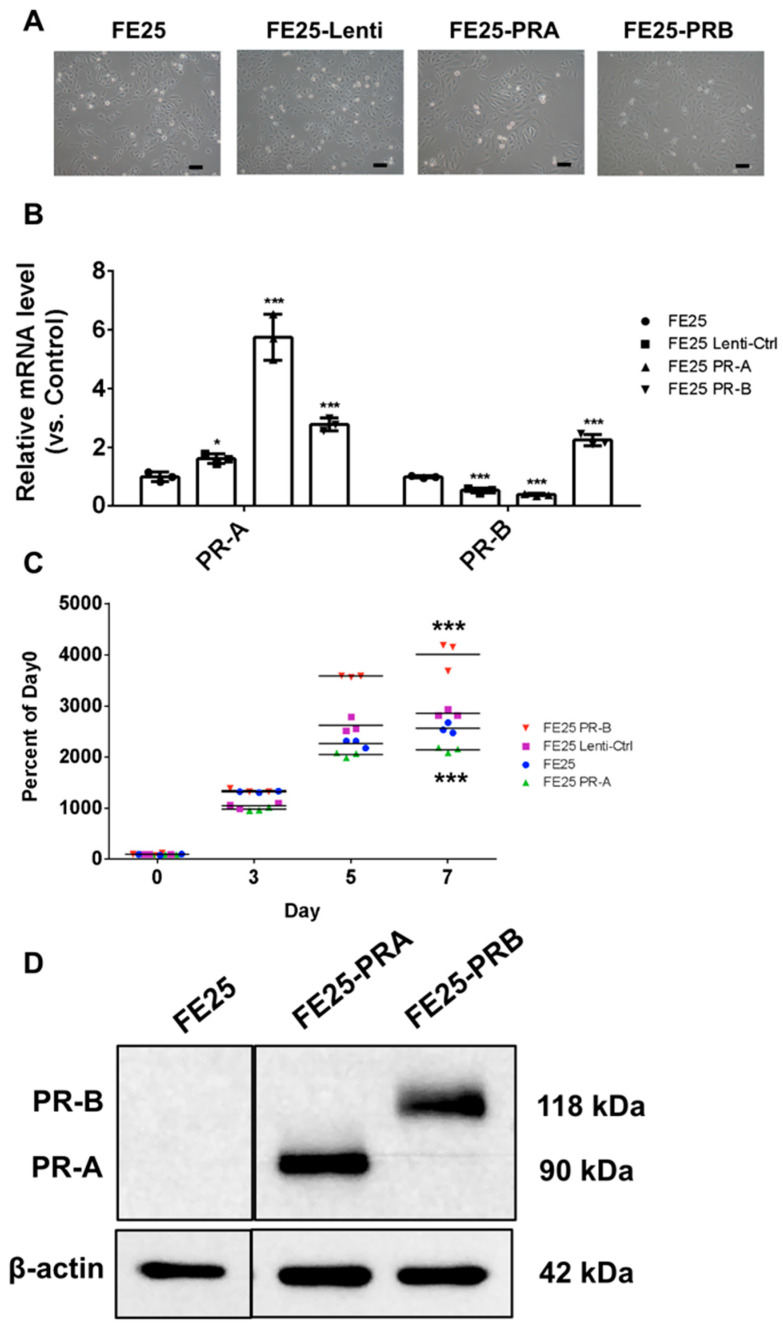
Stable expression of progesterone receptor isoforms PR-A and PR-B in FE25 cells. (**A**) Representative phase-contrast images showing similar cell morphology across parental FE25, lentiviral control (FE25-Lenti), and PR-overexpressing cells (FE25-PRA, FE25-PRB), suggesting no major morphological alterations. (**B**) qRT-PCR analysis confirming successful overexpression of PR-A and PR-B mRNA in FE25-PRA and FE25-PRB cells, respectively, compared with that in the FE25 and FE25-Lenti controls (*n* = 3). (**C**) Cell proliferation assays revealed that PR-A overexpression significantly suppresses FE25 cell growth, whereas PR-B expression significantly enhances proliferation over 7 days (*n* = 3). (**D**) Representative image of Western blot analysis, validating the protein expression of PR-A (90 kDa) and PR-B (118 kDa) in the FE25-PRA and FE25-PRB cells, respectively, with β-actin serving as a loading control (*n* = 3). PR, progesterone receptor; qRT-PCR, quantitative reverse transcription-polymerase chain reaction. * *p* < 0.05, *** *p* < 0.001. One-way ANOVA followed by Bonferroni post hoc testing was used.

Figure 1 illustrates the successful establishment of FE25 cell lines that stably overexpress PR-A and PR-B. Morphologically, all cell groups (FE25, FE25-Lenti, FE25-PR-A, and FE25-PR-B) appeared similar (Figure 1A). Quantitative reverse transcription polymerase chain reaction (qRT-PCR) and Western blotting confirmed the selective overexpression of PR-A and PR-B at both the mRNA and protein levels in the cell lines (Figure 1B,D). Functionally, cell proliferation assays (Figure 1C) revealed that PR-A overexpression significantly suppressed growth, whereas PR-B enhanced proliferation, indicating distinct roles for each isoform in regulating endometrial epithelial cell proliferation.

### 2.2. Differential Effects of PR-A and PR-B Overexpression on Cell Cycle Distribution in FE25 Cells

**Figure 2:**
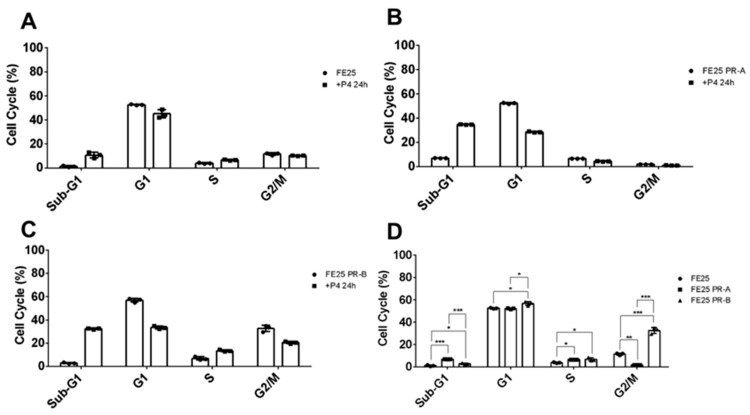
Effects of progesterone (P4) on cell cycle distribution in FE25 cells and cells overexpressing PR-A or PR-B. (**A**) FE25 and P4 (*n* = 3), (**B**) FE25-PR-A and P4 (*n* = 3), (**C**) FE25-PR-B and P4 (*n* = 3), (**D**) FE25, FE25-PR-A, and FE25-PR-B (*n* = 3). The symbol represents each experiment value. * *p* < 0.05, ** *p* < 0.01, *** *p* < 0.001. The Mann–Whitney U test was used.

Figure 2 demonstrates that progesterone (P4) treatment altered cell-cycle distribution in the FE25 cells, with distinct effects depending on PR isoform expression. In parental FE25 cells, P4 induced G1 phase arrest with reduced S phase entry. In the FE25-PR-A cells, P4 markedly increased the Sub-G1 population, indicating apoptosis, and significantly reduced the G1, S, and G2/M phase populations (Figure 2A). FE25-PR-B cells also showed increased Sub-G1 and decreased G1 and G2/M phases after P4 treatment (Figure 2C), although the apoptotic effect was less pronounced than that in the PR-A-expressing cells (Figure 2B). Comparative analysis confirmed that both PR isoforms mediated P4-induced apoptosis and cell-cycle arrest, with PR-A showing stronger pro-apoptotic activity (Figure 2D).

### 2.3. PR Isoforms Differentially Regulate Cell Cycle Inhibitors and Cellular Senescence

**Figure 3:**
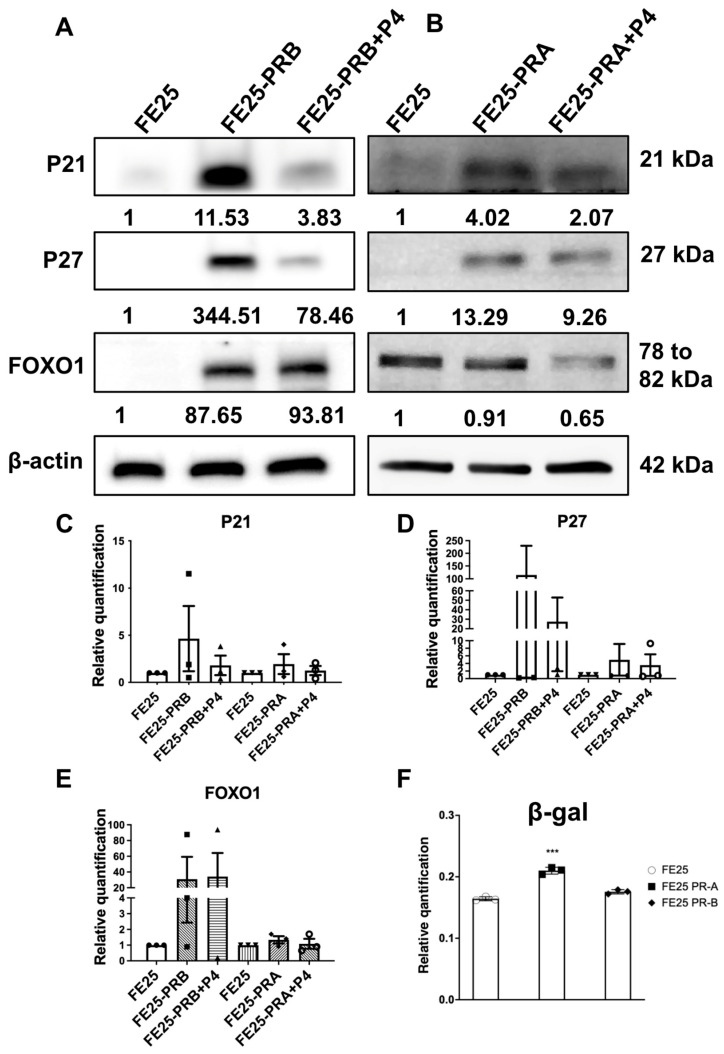
Expression of cell cycle regulators and senescence markers in PR isoform-expressing FE25 cells. (**A**) Representative Western blot analysis showing the expression of P21, P27, and FOXO1 in FE25 and FE25-PR-B cells with or without progesterone (P4) treatment (*n* = 3). Quantification below each blot indicates relative expression levels normalized to those of β-actin. (**B**) Representative Western blot analysis showing the expression of the same proteins in FE25-PR-A cells and the effect of P4 treatment (*n* = 3). (**C**–**E**) Relative quantification of p21, p27, and FOXO1 protein levels normalized to β-actin. Data are shown relative to FE25 controls (*n* = 3). The symbol represents each experiment value. (**F**) β-Galactosidase assay indicating enhanced senescence activity in PR-expressing cells, with FE25-PR-A showing a significant increase (*** *p* < 0.001) compared with FE25 and FE25-PR-B cells (*n* = 3). One-way ANOVA followed by Bonferroni post hoc testing was used.

Western blot analysis revealed distinct effects of PR-A and PR-B on the expression of cell cycle regulators in FE25 cells (Figure 3A,B). In the FE25-PR-B cells, the expression levels of P21, P27, and FOXO1 were markedly upregulated compared with those in parental FE25 cells, indicating the activation of cell-cycle arrest and senescence-related signaling (Figure 3C). Upon P4 treatment, the expression of these proteins was substantially reduced, suggesting ligand-dependent repression of PR-B-induced growth inhibitory pathways (Figure 3C). In contrast, FE25-PR-A cells exhibited only modest induction of P21 and P27 and a slight decrease in FOXO1 levels after P4 stimulation, indicating weaker transcriptional activation of these targets compared with those of PR-B (Figure 3D). Consistent with these findings, the β-galactosidase assays (Figure 3E) demonstrated significantly increased senescence activity in the FE25-PR-A cells compared with that in the FE25 and FE25-PR-B cells (*** *p* < 0.001), confirming that PR isoforms differentially modulated cell-cycle arrest and senescence phenotypes.

### 2.4. Increased Cyclin D1 Expression in PR Isoform-Expressing Cells

**Figure 4:**
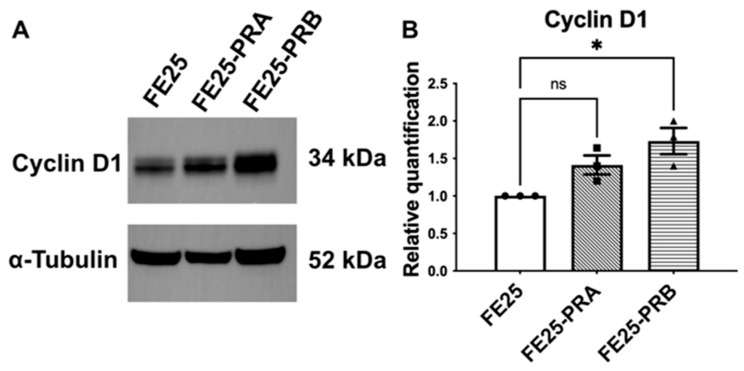
PR-A and PR-B overexpression increase cyclin D1 levels in FE25 cells. (**A**) Representative Western blot showing cyclin D1 protein expression in parental FE25 cells and FE25 cells overexpressing PR-A (FE25-PRA) or PR-B (FE25-PRB) (*n* = 3). α-Tubulin served as the loading control. (**B**) Quantification of cyclin D1 protein levels normalized to α-tubulin and presented relative to FE25 cells (*n* = 3). The symbol represents each experiment value. * *p* < 0.05. ns: no significance.

Overexpression of PR-A and PR-B in FE25 cells led to a marked increase in cyclin D1 protein expression. Western blot analysis demonstrated that both FE25-PR-A and FE25-PR-B cells exhibited higher cyclin D1 levels compared with those in parental FE25 cells, while the α-tubulin levels remained consistent across groups (Figure 4A).

Densitometric quantification confirmed these findings, showing a 1.64-fold increase in cyclin D1 expression in FE25-PRA cells and a 1.80-fold increase in FE25-PRB cells relative to the control FE25 cells (Figure 4B). These results indicate that both PR isoforms enhanced cyclin D1 expression, with PR-B exerting a slightly stronger effect.

### 2.5. Distinct and Shared Transcriptomic Responses to Progesterone in PR-A- and PR-B-Expressing FE25 Cells

**Figure 5:**
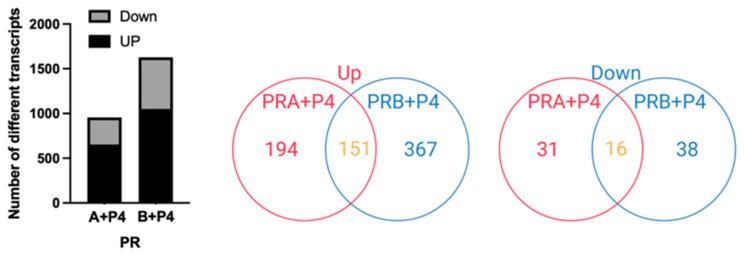
Comparative transcriptomic analysis of progesterone (P4)-treated FE25 cells expressing PR-A or PR-B (*n* = 1 each). (**Left panel**) Bar graph showing the number of significantly upregulated (dark gray) and downregulated (light gray) transcripts in FE25-PR-A and FE25-PR-B cells after P4 treatment, relative to their respective untreated controls. (**Middle panel**) Venn diagram comparing upregulated transcripts in FE25-PR-A+P4 vs. PR-A and FE25-PR-B+P4 vs. PR-B groups, identifying 151 commonly upregulated genes (yellow color). (**Right panel**) Venn diagram showing overlapping downregulated transcripts between the two treatments, with 16 genes shared by both PR-A and PR-B conditions (yellow color).

Figure 5 illustrates the differential transcriptomic responses to P4 treatment in FE25 cells expressing PR-A or PR-B. The bar graph (left) shows that the P4 treatment resulted in a greater number of differentially expressed transcripts in the FE25-PR-B cells (over 1600 genes) than in the FE25-PR-A cells (approximately 900 genes), with a higher proportion of upregulated genes in both groups. The Venn diagrams (middle and right) revealed 151 upregulated and 16 downregulated genes shared between the PR-A+P4 and PR-B+P4 conditions, while the majority of transcriptional changes were unique to each PR isoform. These findings indicate that P4 induced both overlapping and isoform-specific gene expression patterns, with a more robust transcriptomic response observed in PR-B-expressing cells.

### 2.6. P4-Induced Downregulation of PI3K–Akt and MAPK Signaling Pathways in PR-A and PR-B-Expressing FE25 Cells

**Figure 6:**
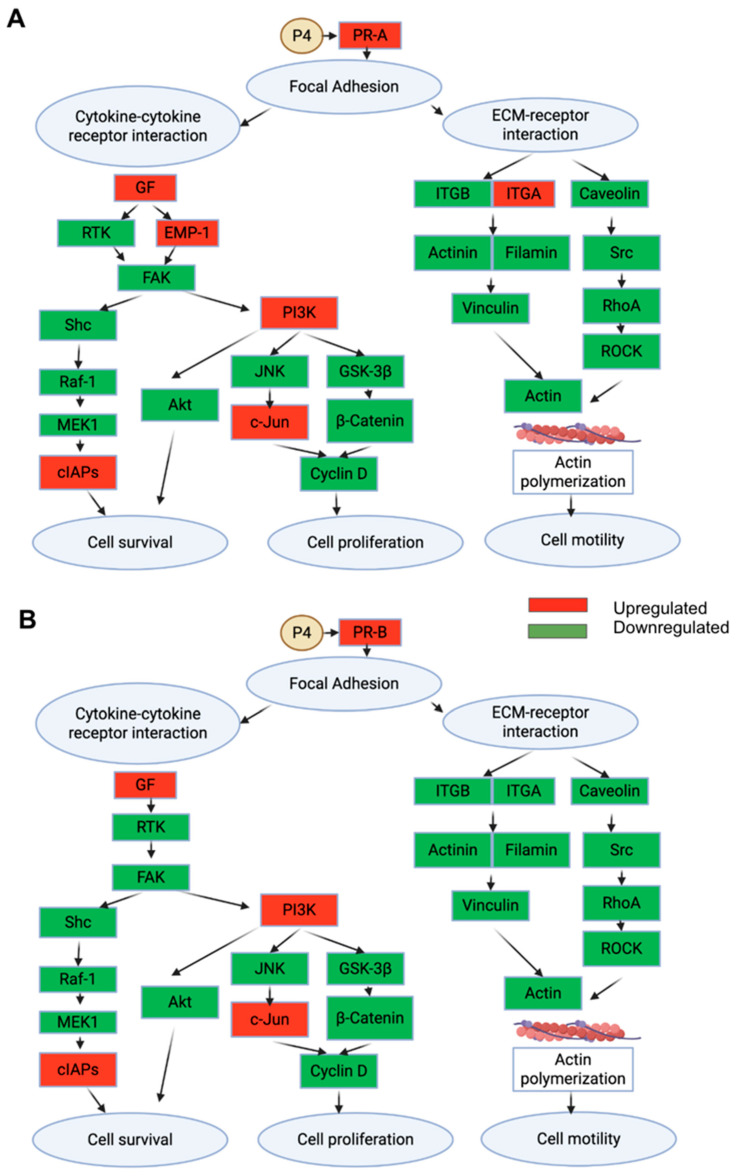
Pathway enrichment analysis of progesterone (P4) treatment in PR isoform-expressing FE25 cells. Gene expression in (**A**) FE25-PR-A cells treated with P4 versus the untreated FE25-PR-A cells (*n* = 1 each) and in (**B**) FE25-PR-B cells treated with P4 versus the untreated FE25-PR-B cells (*n* = 1 each).

Figure 6 presents pathway enrichment maps highlighting the effects of P4 treatment on FE25 cells overexpressing PR-A and PR-B, specifically comparing the P4-treated cells to their untreated counterparts. Figure 6A shows that in FE25-PR-A cells, P4 treatment downregulates multiple signaling nodes (marked in green) within focal adhesion, the PI3K–Akt, and MAPK pathways—such as PI3K, c-Jun, and eIF4EBP1—indicating suppression of cell proliferation and survival pathways. Meanwhile, a few elements, such as ITGA/ITGB and RTK, were upregulated (red), suggesting selective activation. Figure 6B similarly shows that in the FE25-PR-B cells, P4 downregulates components of the same pathways (e.g., PI3K, SOS, FAK), though with slightly fewer nodes suppressed than in the PR-A condition. These pathway maps suggest that P4 treatment broadly inhibits proliferative and survival signaling cascades in both the PR-A- and PR-B-expressing cells, with PR-A showing greater pathway suppression.

### 2.7. Differential Activation of MAPK and PI3K Signaling by PR Isoform

**Figure 7:**
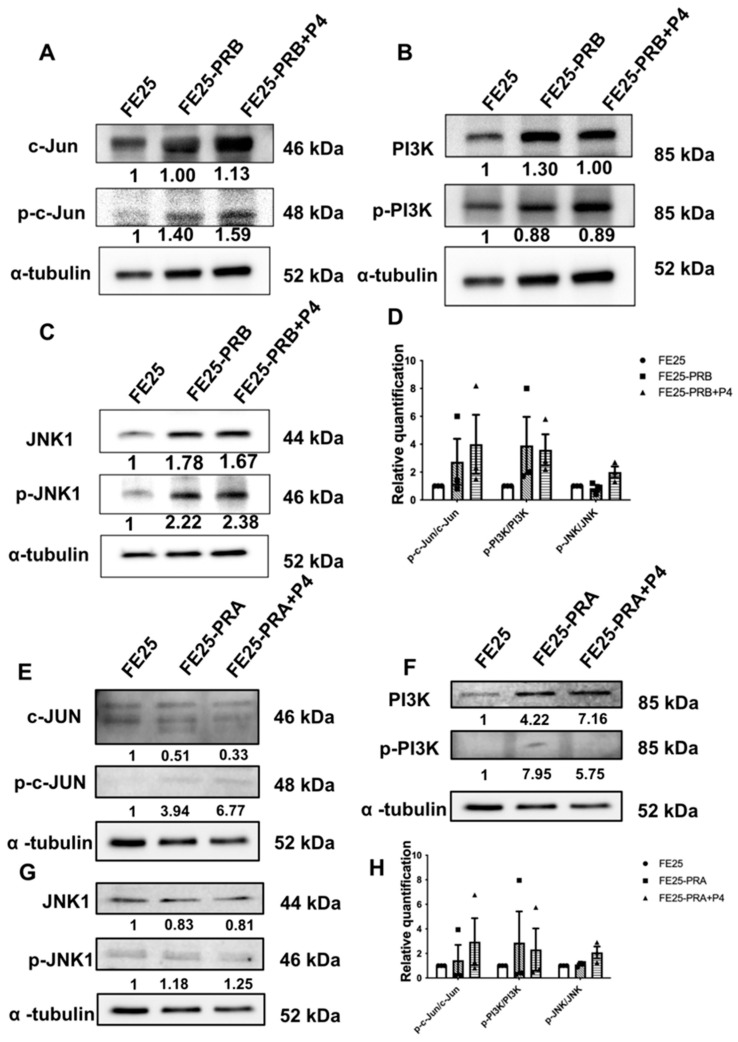
PR isoforms differentially modulate the c-Jun, JNK, and PI3K signaling pathways in FE25 cells. (**A**–**C**) Western blot analysis showing the expression of c-Jun, phosphorylated c-Jun (p-c-Jun), PI3K, phosphorylated PI3K (p-PI3K), JNK1, and phosphorylated JNK1 (p-JNK1) in FE25 and FE25-PR-B cells with or without progesterone (P4) treatment (*n* = 3). (**D**) Relative quantification of p-c-Jun, p-PI3K, and p-JNK1 protein levels normalized to total protein in (**A**–**C**). Data are shown relative to the FE25 controls. The symbol represents each experiment value. (**E**–**G**) Western blot analysis of the same signaling molecules in FE25-PR-A cells (*n* = 3). (**H**) Relative quantification of p-c-Jun, p-PI3K, and p-JNK1 protein levels normalized to total protein in (**A**–**C**). The symbol represents each experiment value. Data are shown relative to the FE25 controls.

Western blot analysis demonstrated distinct effects of PR-A and PR-B on MAPK and PI3K pathway activation in the FE25 cells (Figure 7). In the FE25-PR-B cells, phosphorylation of c-Jun and JNK1 was markedly increased compared with that in the FE25 parental cells, indicating activation of the JNK signaling cascade (Figure 7A,C,D). P4 treatment further enhanced c-Jun phosphorylation without significantly affecting PI3K phosphorylation (Figure 7B,D), suggesting that PR-B primarily promotes JNK/c-Jun signaling in a ligand-independent manner. Conversely, FE25-PR-A cells exhibited a different activation pattern, characterized by elevated p-c-Jun and p-PI3K levels, particularly after P4 treatment, while the total c-Jun expression was suppressed (Figure 7E,F,H). Phosphorylation of JNK1 remained largely unchanged in the PR-A cells (Figure 7G,H). These results indicate that PR-B preferentially activated the JNK/c-Jun axis, whereas PR-A mainly enhanced PI3K signaling, highlighting isoform-specific modulation of intracellular signaling pathways in ovarian epithelial cells.

### 2.8. P4-Induced Transcriptomic Shifts in Immune Response, Proliferation, and Growth Regulation in FE25-PR-A Cells

**Figure 8:**
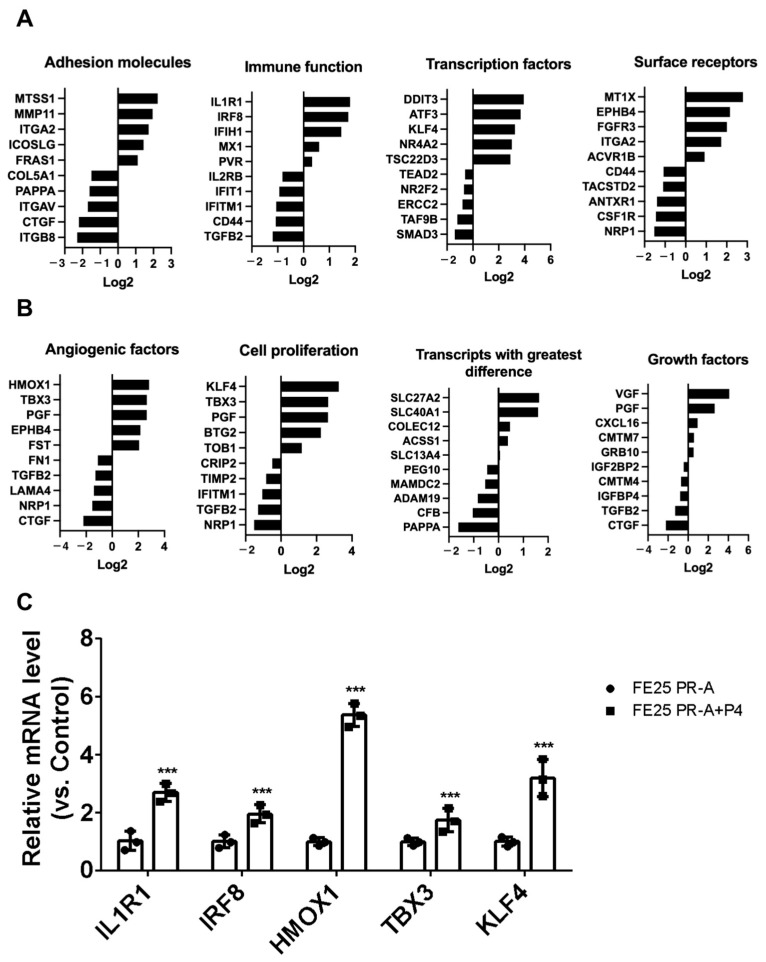
Differential gene expression profiles in FE25-PR-A cells with and without progesterone (P4) treatment (*n* = 1 each). (**A**) Selected gene categories showing differential expression between the untreated FE25-PR-A and P4-treated FE25-PR-A cells, including genes involved in adhesion molecules, immune function, transcription factors, and surface receptors. Log2 fold change values are plotted to indicate relative expression, with positive values favoring the P4-treated group and negative values favoring the untreated group. (**B**) Additional functional categories highlighting differences in genes related to angiogenic factors, cell proliferation, transcripts with the greatest difference, and growth factors between the FE25-PR-A and P4-treated FE25-PR-A cells. (**C**) qPCR validation of selected differentially expressed genes in FE25 PR-A cells with or without P4 treatment (*n* = 3). Relative mRNA expression levels of IL1R1, IRF8, HMOX1, TBX3, and KLF4 were measured in the FE25 PR-A cells (black bars) and FE25 PR-A cells treated with P4 (gray bars), and normalized to control levels. Data are presented as the mean ± SEM from three independent experiments (*n* = 3). The symbol represents each experiment value. *** *p* < 0.001 compared with the FE25 PR-A control.

Figure 8 illustrates the transcriptional changes induced by P4 treatment in FE25-PR-A cells, highlighting key functional gene categories. Figure 8A shows that P4 treatment upregulates genes involved in immune function (e.g., IL1R1, IRF8, IFIH1), surface receptors (e.g., FGFR3, MT1X), and specific transcription factors (e.g., DDIT3, ATF3) while downregulating several adhesion molecules (e.g., CTGF, ITGB8) and pro-inflammatory genes. Figure 8B further revealed the P4-mediated upregulation of genes related to cell proliferation (e.g., KLF4, TBX3), angiogenesis (e.g., HMOX1, TBX3), and growth factors (e.g., VGF, PGF, CXCL16), while several proliferation-associated transcripts (e.g., NRP1, TGFB2, and IFITM1) were more highly expressed in the untreated FE25-PR-A group. qRT-PCR analysis confirmed that P4 treatment significantly upregulated IL1R1, IRF8, HMOX1, TBX3, and KLF4 expression in the FE25 PR-A cells compared with the untreated controls (Figure 8C). These results suggest that progesterone exerts a broad regulatory effect on genes associated with immune modulation, cell proliferation, and tissue remodeling in PR-A-expressing precancerous FTECs.

### 2.9. P4-Induced Gene Expression Changes Highlight Immune, Proliferative, and Angiogenic Shifts in FE25-PR-B Cells

**Figure 9:**
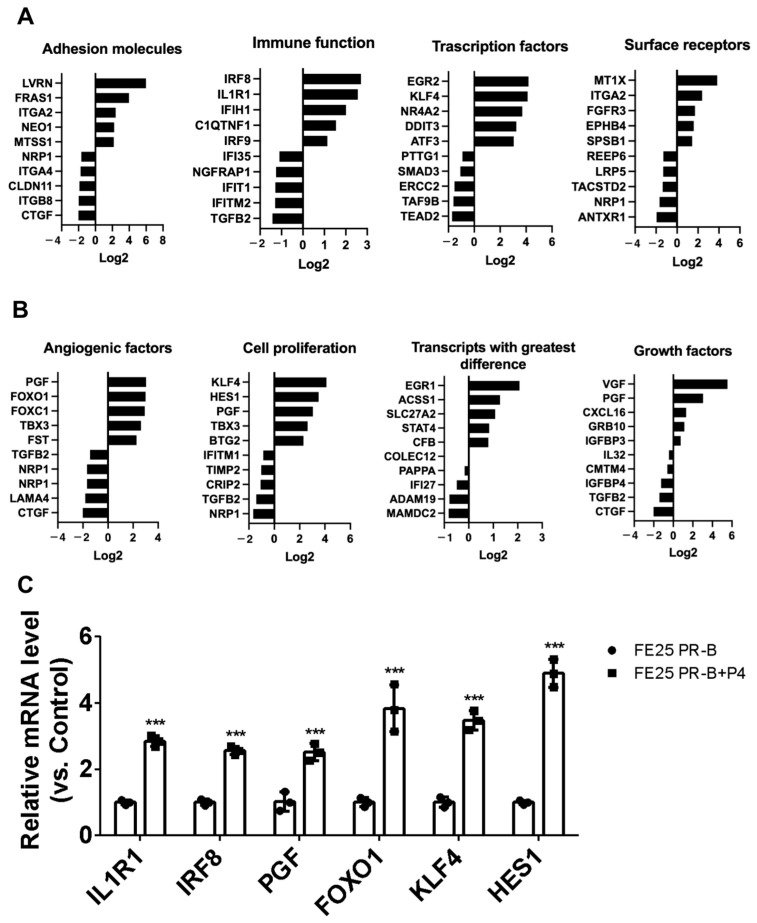
Transcriptomic profiling of FE25-PR-B cells following progesterone (P4) treatment (*n* = 1 each). (**A**) Differentially expressed genes between untreated FE25-PR-B and P4-treated FE25-PR-B cells are grouped by functional categories, including adhesion molecules, immune function, transcription factors, and surface receptors. Log2 fold changes indicate upregulation (positive values) or downregulation (negative values) in response to P4. (**B**) Additional comparisons shown for genes related to angiogenic factors, cell proliferation, transcripts with the greatest expression differences, and growth factors. (**C**) qPCR validation of selected differentially expressed genes in FE25 PR-B cells with or without P4 treatment. Relative mRNA expression levels of IL1R1, IRF8, PGF, FOXO1, KLF4, and HES1 were measured in the FE25 PR-B cells (black bars) and FE25 PR-B cells treated with P4 (gray bars) and normalized to the control levels. Data are presented as the mean ± SEM from three independent experiments (*n* = 3). The symbol represents each experiment value. *** *p* < 0.001 compared with FE25 PR-B control.

Figure 9 presents the transcriptomic changes in FE25-PR-B cells following P4 treatment, highlighting key functional gene categories. Figure 9A shows that P4 upregulated genes related to immune function (e.g., IL1R1, IRF8, IFIH1), surface receptors (e.g., FGFR3, MT1X, IRGA2), and transcription factors (e.g., EGR2, KLF4) while downregulating adhesion molecules (e.g., CLDN11, ITGB8, CTGF) and several immune regulators. Figure 9B further illustrates the increased expression of angiogenic and growth factors (e.g., PGF, FOXO1, FOXC1) and proliferation-related genes (e.g., KLF4, EGFR, PGF) in P4-treated cells. Several transcripts (e.g., EGR1, STAT4, ACSS1) showed the greatest fold differences. qRT-PCR analysis confirmed that P4 treatment significantly upregulated IL1R1, IRF8, PGF, FOXO1, KLF4, AND HES1 expression in the FE25 PR-B cells compared with the untreated controls (Figure 9C). These results suggest a broad regulatory role of PR-B activation by P4 in modulating immune response, angiogenesis, and cellular proliferation in precancerous FTECs.

## 3. Discussion

Collectively, the study findings demonstrate that PR isoforms, PR-A and PR-B, exert distinct regulatory effects on proliferation, apoptosis, senescence, and transcriptomic signaling in FE25 precancerous FTECs. Stable overexpression of PR-A and PR-B was successfully established and verified at both the mRNA and protein levels, with PR-A suppressing cell proliferation and PR-B enhancing it. Upon P4 treatment, both isoforms induced cell-cycle arrest and apoptosis, but PR-A induced a markedly stronger apoptotic Sub-G1 response than PR-B. PR isoforms also differentially regulated cell-cycle inhibitors and senescence markers: PR-B strongly upregulated them, leading to increased expression of p21, p27, and FOXO1—effects attenuated by P4—whereas PR-A induced robust β-galactosidase activity, indicating enhanced senescence.

Transcriptomic analyses further revealed broad, isoform-dependent shifts in gene expression following P4 treatment, with PR-B eliciting a larger number of differentially expressed genes than PR-A. Both isoforms showed overlapping but distinct activation patterns in genes related to immune regulation, proliferation, angiogenesis, and tissue remodeling. Pathway enrichment analysis demonstrated differential modulation of focal adhesion, PI3K–Akt, and MAPK signaling, with PR-A and PR-B each suppressing proliferative pathways upon P4 exposure but activating unique signaling nodes in the absence of ligands. These integrated results highlight the divergent yet complementary roles of PR-A and PR-B in mediating P4-dependent regulation of cellular growth, senescence, and transcriptional reprogramming in FTECs.

PR-A can promote migration and invasion. However, its activity tends to restrain proliferation and induce cell-cycle exit, partly by modulating the DREAM complex and the FOXO1/p21 axis, leading to senescence and apoptosis in p53-mutant ovarian epithelial models [6,21,22]. In contrast, PR-B is linked to proliferation enhancement. PR-B functions as the dominant ligand-dependent isoform, driving cell-cycle progression and mitotic entry by upregulating genes involved in proliferation, including those in the AKT signaling pathway [6]. Overexpression of PR-B in ovarian tissue models results in increased cell proliferation and tumorigenesis, with transcriptomic profiles resembling aggressive ovarian and endometrial cancers [6]. PR-B also enhances platinum sensitivity and apoptosis in HGSC, suggesting a complex role in both tumor growth and therapeutic response [15]. Our study also found that PRB enhanced cell proliferation and upregulated the FOXO1/p21 signaling pathway.

Mechanistically, PR-A is closely associated with growth suppression, apoptosis, and senescence by coordinating the modulation of multiple signaling pathways. PR-A reduces PI3K–Akt and MAPK activity while enhancing FOXO1 function and upregulating the cell-cycle inhibitors p21 and p27 [17]. Although PR-A can repress FOXO1 and p21 under certain conditions, the presence of active FOXO1 allows PR-A to cooperate in driving senescence and G1 cell-cycle arrest. Attenuation of PI3K–Akt signaling prevents FOXO1 phosphorylation and degradation, promoting its nuclear localization and the transcriptional activation of p21 and p27, which in turn induces G1 arrest and senescence. Concurrent inhibition of MAPK/JNK further decreases cyclin D1 expression, reinforcing cell cycle exit [23]. Overall, PR-A predominantly regulates genes in a largely progestin-independent manner, leading to increased expression of cell-cycle inhibitors and pro-apoptotic factors, culminating in growth inhibition, apoptosis, and senescence [22,24]. Consistent with these mechanisms, our findings support previous reports that PR-A suppresses proliferation by inducing FOXO1-dependent expression of p21 and p27.

In contrast, PR-B enhances proliferation by activating the PI3K–Akt and MAPK pathways, which inactivate FOXO1 via phosphorylation, reduce p21/p27, and upregulate cyclin D1 [25]. PR-B is the dominant ligand-dependent isoform, and its activation leads to the robust induction of genes promoting cell-cycle progression and mitosis, including cyclin D1 [26]. Enhanced PI3K–Akt signaling downstream of PR-B results in FOXO1 inactivation and cytoplasmic sequestration, thereby reducing its tumor-suppressive effects. MAPK pathway activation by PR-B also promotes cyclin D1 expression and cell proliferation [27]. Our findings suggest that PR-B promotes growth by activating other pathways such as c-Jun and JNK1.

The proliferative phenotype of PR-B observed in the absence of ligand in our overexpression model should not be interpreted as contradicting the favorable prognostic association of PR-B in clinical HGSC. Rather, these observations highlight that PR-B function is determined by both ligand occupancy and cellular context. Ligand-independent PR-B signaling drives proliferation via JNK/c-Jun cross-talk, whereas progesterone-activated PR-B reprograms transcription toward growth arrest and apoptosis—effects that likely underlie the improved prognosis and platinum sensitivity observed in PR-B-positive tumors clinically. The mechanistic distinctions identified in vitro should not be extrapolated as direct predictors of clinical prognosis or treatment response.

In FE25 cells, which are characterized by p53 and Rb inhibition, PR-B overexpression enhances basal proliferation, likely reflecting ligand-independent PR-B signaling and its cross-talk with mitogenic pathways in the absence of intact cell-cycle checkpoints [28]. In contrast, progesterone binding reprograms PR-B activity toward a ligand-dependent transcriptional program, leading to growth arrest and apoptosis [29]. This context-dependent switch reconciles the apparent paradox and highlights that PR-B function is determined by both ligand status and tumor suppressor background, underscoring the complexity of progesterone signaling in fallopian tube-derived cancer models.

Our findings in FE25 cells provide mechanistic insight relevant to early HGSC pathogenesis, as these cells recapitulate key features of precursor lesions with p53 and Rb dysfunction [18]. In this context, the balance between PR isoforms appears critical in determining cellular responses to progesterone, with PR-B promoting basal proliferation in a ligand-independent manner, while ligand-activated PR signaling shifts toward growth suppression and apoptosis [6]. This isoform-dependent switch may determine whether early fallopian tube lesions expand or are eliminated following progesterone exposure. Clinically, such differences in PR isoform composition could help explain heterogeneity in progesterone responsiveness observed in HGSC [15]. These suggest that PR isoform status in early lesions may modulate tumor-initiation risk and the therapeutic or preventive efficacy of progesterone-based strategies.

PR expression has been associated with a favorable prognosis in some clinical HGSC cohorts [8]. The PR isoform-specific effects observed in the FE25 model under progesterone stimulation do not fully mirror these population-level survival associations [8]. This discrepancy likely reflects differences between simplified in vitro systems and the complex clinical context, where PR status integrates tumor heterogeneity, stromal interactions, treatment exposure, and disease stage. Our findings suggest that PR-B preferentially engages apoptosis-related signaling, whereas PR-A is more closely linked to growth arrest and senescence. However, these mechanistic distinctions should not be interpreted as direct predictors of chemotherapy response or patient survival.

A limitation of this study is that PR isoform-dependent apoptotic signaling under progesterone exposure does not directly correlate with platinum chemosensitivity. A limitation of this study is the lack of isoform-specific loss-of-function experiments (e.g., siRNA/shRNA knockdown or CRISPR-mediated depletion), which are technically challenging given the shared genomic locus of PR-A and PR-B. Future studies employing isoform-selective knockdown strategies will be important to corroborate the causal interpretation of the gain-of-function phenotypes observed here. Because 100 μM progesterone represents a pharmacologic concentration exceeding the physiologic serum levels, our findings reflect mechanistic effects under in vitro conditions and should be interpreted with caution when extrapolating to in vivo or clinical settings. Dose–response experiments using lower, physiologically relevant progesterone concentrations would be informative in future studies to delineate the concentration dependency of PR isoform-specific effects. A limitation of this study is the absence of an empty vector control across all downstream functional and protein analyses, which precludes the complete exclusion of potential non-specific lentiviral transduction effects on the observed phenotypes. Similarly, the absence of a vehicle-only control for progesterone treatment precludes the complete exclusion of potential solvent-related contributions to the observed cellular responses. Future experiments should incorporate both controls in all assay conditions. A significant limitation of this study is that RNA-Seq was performed with *n* = 1 per condition, which is insufficient for formal statistical differential expression analysis and precludes the use of standard replicate-based DEG calling algorithms with controlled FDR. These transcriptomic data should therefore be interpreted as exploratory and hypothesis-generating. The qRT-PCR validation of selected transcripts in three independent biological replicates provides partial corroboration, but future studies with fully replicated RNA-Seq (minimum *n* = 3 per condition) are required to establish statistical robustness. Although PI3K–Akt and MAPK pathway modulation was corroborated by phosphoprotein Western blot analysis, formal pathway inhibition experiments and transcriptional reporter assays were not performed. Future studies employing selective inhibitors of PI3K, JNK, or MAPK in the PR-A and PR-B overexpression backgrounds, combined with functional readouts of apoptosis and senescence, would further strengthen the causal attribution of these pathways to the observed phenotypes. All experiments were conducted in a single FE25 cell line, which, while representing a well-characterized precancerous FTEC model with HGSC-relevant molecular features, limits the generalizability of the findings. Validation in additional fallopian tube epithelial and ovarian cancer models, including organoid systems and PR-reconstituted HGSC lines, will be important to confirm that the isoform-specific biological and transcriptomic effects observed here are broadly applicable rather than cell-line specific.

## 4. Materials and Methods

### 4.1. Cell Lines

The FE25 cell line (P53 and RB inhibited) was used in the experiments [6]. The cells were cultured in MCDB105 and M199 medium (Sigma, St. Louis, MO, USA) plus 10% fetal bovine serum, 100 IU/mL penicillin, and 100 μg/mL streptomycin. Cultures were maintained in 75 cm^2^ flasks with 12 mL of medium and incubated at 37 °C in a humidified atmosphere containing 5% CO_2_.

### 4.2. Overexpression of PR in FE25

FE25 cells were maintained under standard culture conditions (37 °C, 5% CO_2_) and used at 50–70% confluence for transduction. Human PR-A and PR-B cDNAs were individually cloned into separate third-generation lentiviral expression vectors driven by a cytomegalovirus promoter and containing a puromycin-resistance cassette (ABM Company, Richmond, BC, Canada). Lentiviral particles were generated by co-transfecting HEK293T cells with the PR-A or PR-B expression plasmids together with psPAX2 and pMD2.G packaging plasmids using Lipofectamine 3000. Lentiviral stocks were concentrated by ultracentrifugation, and viral titers (2 × 10^8^ TU/mL) were quantified prior to use. Cells were infected at the indicated MOI (=5) to ensure comparable transduction efficiency across experiments. Viral supernatants were harvested at 48 h and 72 h, cleared through a 0.45-µm filter, and concentrated using ultracentrifugation (25,000× *g*, 2 h, 4 °C). FE25 cells were transduced with either PR-A- or PR-B-expressing lentivirus at a multiplicity of infection of 5–10 in the presence of 8 µg/mL polybrene. After 24 h, the media were refreshed, and the cells were recovered for an additional 48 h before puromycin selection (1–2 µg/mL for 5–7 days). Stable FE25-PR-A and FE25-PR-B lines were expanded, and the overexpression of each isoform was verified through qRT-PCR using isoform-specific primers and Western blotting using antibodies discriminating PR-A and PR-B.

### 4.3. Proliferation of Cells

Cell proliferation was tested using the XTT assay (2,3-bis(2-methoxy-4-nitro-5-sulfophenyl)-5-[(phenylamino) carbonyl]-2H-tetrazolium hydroxide). The tested cells were seeded in a 96-well plate at a density of 2 × 10^3^ cells in 100 μL of culture medium. The optical density of the cells was measured at days 0, 3, 5, and 7. The tested cells were incubated with 150 μL XTT solution (Biological Industries, Beit-Haemek, Israel) for 3 h at 37 °C in an incubator. The microplate reader (Bio-Rad Model 3550, Hercules, CA, USA) was used to measure the optical density at 450 nm. The optical densities at each time point were used to construct proliferation curves for the tested cell lines.

### 4.4. Cell Cycle Analysis

To evaluate P4-induced cell cycle arrest and apoptosis mediated by PR isoforms, FE25, FE25-PR-A, and FE25-PR-B cells were seeded at 60–70% confluence and treated with P4 (100 μM) or the vehicle control for 24 h. Following treatment, cells were harvested, washed with cold PBS, and fixed in 70% ethanol at −20 °C overnight. For cell-cycle analysis, fixed cells were incubated with RNase A (100 µg/mL, EN0531, ThermoFisher, Waltham, MA, USA) and stained with propidium iodide (PI, 50 µg/mL, P1304MP, ThermoFisher) for 30 min in the dark. DNA content was measured using a flow cytometer, and cell populations in the Sub-G1, G1, S, and G2/M phases were quantified. The Sub-G1 fraction was used as an indicator of apoptosis. Data were analyzed using FlowJo software (version 10, BD Science, Franklin Lakes, NJ, USA) to compare P4-induced changes across the parental FE25, FE25-PRA, and FE25-PRB cells. All experiments were performed in triplicate.

### 4.5. Extraction of Total RNA from Cells

In this experiment, total RNA was extracted using the RNeasy Mini Kit (QIAGEN, Hilden, Germany). Cells (5 × 10^5^) were seeded in a 10-cm culture dish and cultured for 24 h. After removing the culture medium, the cells were washed twice with 1× PBS, treated with 0.05% trypsin, and collected. Subsequently, cell pellets were lysed in 700 μL RLT buffer, and the solution was aspirated several times with a 1 mL micropipette until the cells were completely dissolved and the solution became transparent. The reagents were added to extract RNA according to the manufacturer’s instructions. The extracted RNA samples were stored at −80 °C in the refrigerator until use, and their concentrations were measured.

### 4.6. cDNA Preparation

cDNA was prepared using the Reverse-iTTM 1st Strand Synthesis Kit (ABgene). Briefly, 1 μg of total RNA was mixed with 1 μL of anchored oligo(dT) and supplemented with diethylpyrocarbonate-treated water to a final volume of 12 μL. The mixture was incubated at 70 °C for 5 min and chilled on ice. An 8 μL reaction mixture (4 μL 5× First-strand synthesis buffer, 2 μL dNTP mix [5 mM each], 1 μL 100 mM DTT, and 1 μL Reverse-iTTM RTase blend) was added, and the reaction was incubated at 47 °C for 50 min. The reaction was terminated at 75 °C for 10 min, and the prepared cDNA was stored at −20 °C in a refrigerator until use.

### 4.7. RNA Sequencing

Gene expression profiling was conducted for the FE25, FE25-PR-A, and FE25-PR-B cells. The original data were obtained using high-throughput sequencing (Illumina NovaSeq 6000 platform) and contained 28,264 human genome probes. Up- and downregulated genes in FE25-PR-A or PR-B compared with FE25 were identified and subjected to Gene Ontology analysis using gene set enrichment analysis (GSEA) [26]. The online GSEA (https://www.gsea-msigdb.org/gsea/index.jsp, accessed on 1 April 2025.) was used to identify functional gene sets with a significant false detection rate (FDR) q-value < 0.05.

### 4.8. qRT-PCR

Total RNA was isolated using TRIzol reagent (Invitrogen) following the manufacturer’s protocol, and RNA concentration and purity were assessed spectrophotometrically. One microgram of total RNA was reverse-transcribed into cDNA using a high-capacity reverse transcription kit. Quantitative real-time PCR (qRT-PCR) was performed using SYBR Green Master Mix (ROX, Basel, Switzerland) on a real-time PCR detection system [ABI Step One Plus system (Applied Biosystems, Waltham, MA, USA)] with gene-specific primers (Table 1). Thermal cycling conditions consisted of an initial denaturation step followed by 40 amplification cycles. Melt curve analysis was conducted to confirm primer specificity. Relative gene expression levels were normalized to GAPDH as an internal control and calculated using the 2^−ΔΔCt^ method. All experiments were performed in technical triplicate with at least three independent biological replicates.

### 4.9. Kyoto Encyclopedia of Genes and Genomes Pathway Analysis

Differentially expressed genes identified from experimental comparisons were subjected to Kyoto Encyclopedia of Genes and Genomes (KEGG) pathway enrichment analysis. Gene lists with corresponding fold-change and adjusted *p*-values were analyzed using the clusterProfiler package in R (version 4.3). Enrichment significance was determined using a hypergeometric test, with Benjamini–Hochberg correction applied to control the FDR. Pathways with FDR < 0.05 were considered significantly enriched. KEGG pathway maps were visualized and annotated using KEGG Mapper. Enrichment scores were calculated as –log10(FDR) for ranking and visualization. Pathway maps were additionally annotated using KEGG Mapper to illustrate the distribution of up- and downregulated genes within individual signaling pathways.

### 4.10. Western Blot Analysis

FE25, FE25-PR-A, and FE25-PR-B cells were cultured under standard conditions and harvested for protein extraction. To evaluate downstream signaling of PR isoforms, cells were treated with P4 (100 μM) or the vehicle control for 48 h prior to lysis. Total protein was extracted using RIPA buffer supplemented with protease and phosphatase inhibitors, and protein concentrations were quantified using the bicinchoninic acid assay. Equal amounts of protein (20–30 μg) were resolved on 8–12% sodium dodecyl sulfate-polyacrylamide gels and transferred onto polyvinylidene fluoride membranes. After blocking with 5% non-fat milk in tris-buffered saline with Tween-20 for 1 h at 25 °C membranes were incubated overnight at 4 °C with primary antibodies against PR-A/B (1:1000, #8757; Cell Signaling Technology, Danvers, MA, USA), FOXO1, cyclin D1, p21, p27, c-Jun, JNK, and PI3K (all antibodies purchased from Cell Signaling, Danvers, MA, USA). After washing, membranes were incubated with horseradish peroxidase-conjugated secondary antibodies (Abclonal, Woburn, MA, USA) for 1 h at room temperature. Protein signals were visualized by enhanced chemiluminescence and quantified by densitometry in ImageJ (version 1.54g, National Institutes of Health, Bethesda, MD, USA). β-Actin (#4970, Cell Signaling) or α-tubulin (#2144, Cell Signaling, 1:10,000) served as loading controls. Expression levels in FE25, FE25-PR-A, and FE25-PR-B cells—with and without P4 treatment—were compared to assess the isoform-specific regulation of downstream targets. Western blot signals were quantified by densitometry using ImageJ; phosphorylated protein levels were normalized to their corresponding total protein levels, and all values were further normalized to the indicated loading control.

### 4.11. Quantitative β-Galactosidase Activity Assay

β-Galactosidase activity was quantitatively assessed in the FE25, FE25-PR-A, and FE25-PR-B cells using a colorimetric β-galactosidase activity assay kit (Abcam, Cambridge, MA, USA), following the manufacturer’s instructions. Cells were seeded in 96-well plates and treated under experimental conditions until reaching 70–80% confluence. Cells were lysed, and equal volumes of cell lysate were incubated with the β-galactosidase substrate solution at 37 °C for the recommended incubation period. Enzymatic activity was quantified by measuring absorbance at 420 nm using a microplate reader. Background absorbance from blank wells was subtracted from all readings. Optical density values were normalized to protein concentration measured through the bicinchoninic acid assay. Relative β-galactosidase activity among the FE25, FE25-PR-A, and FE25-PR-B cells was compared to determine isoform-dependent effects on senescence induction.

### 4.12. Comparative Transcriptomic Analysis of P4-Treated FE25-PR-A and FE25-PR-B Cells

To identify shared and isoform-specific transcriptional responses, gene lists of upregulated and downregulated differentially expressed genes from the two comparisons were analyzed using VennDiagram (R package) to generate Venn diagrams illustrating the overlap between PR-A- and PR-B-mediated responses. Upregulated and downregulated gene sets common to both isoforms were extracted for downstream functional annotation. All analyses were conducted using R (version 4.3), and graphical outputs were generated in RStudio (version 2025.05, Posit, PBC, Boston, MA, USA).

### 4.13. Statistical Analyses

Data are expressed as the mean ± standard deviation from at least three independent experiments. Comparisons between the two groups were performed using the Mann–Whitney U test, while comparisons among three or more groups were assessed using one-way ANOVA followed by Bonferroni post hoc testing. Both tests were two-tailed. Statistical analyses were conducted using GraphPad Prism 6 (La Jolla, CA, USA). A *p*-value < 0.05 was considered statistically significant.

## 5. Conclusions

This study demonstrates that PR isoforms PR-A and PR-B exert fundamentally different yet complementary biological effects in precancerous FE25 FTECs. PR-A functions predominantly as a growth-suppressive isoform, limiting proliferation, enhancing apoptosis, and promoting cellular senescence, particularly upon exposure to P4. In contrast, PR-B enhances basal cell proliferation and activates distinct signaling pathways, yet remains responsive to P4-induced growth inhibition. Transcriptomic profiling further revealed that each isoform mediates distinct patterns of immune, angiogenic, and proliferative gene regulation, with PR-B exhibiting a broader transcriptional response than PR-A. Together, these findings highlight the isoform-specific roles of PR-A and PR-B in modulating P4 signaling, cell-cycle control, and cellular stress responses, underscoring their relevance in the early molecular events of fallopian tube epithelial transformation and hormone-driven tumorigenesis.

## Figures and Tables

**Table 1:** Primer sequence.

Gene	Forward Primers (5′->3′)	Reverse Primers (5′->3′)	Product Size (bp)
*IL1R1*	GTGCTTTGGTACAGGGATTCCTG	CACAGTCAGAGGTAGACCCTTC	121
*IRF8*	AGGTCTTCGACACCAGCCAGTT	GCACGAGAATGAGTTTGGAGCG	144
*HMOX1*	CCAGGCAGAGAATGCTGAGTTC	AAGACTGGGCTCTCCTTGTTGC	144
*TBX3*	GGACACTGGAAATGGCCGAAGA	GCTGCTTGTTCACTGGAGGACT	123
*KLF4*	CCCACACAGGTGAGAAACCT	ATGTGTAAGGCGAGGTGGTC	169
*PGF*	GGCGATGAGAATCTGCACTGTG	ATTCGCAGCGAACGTGCTGAGA	127
*FOXO1*	CTACGAGTGGATGGTCAAGAGC	CCAGTTCCTTCATTCTGCACACG	138
*HES1*	GGAAATGACAGTGAAGCACCTCC	GAAGCGGGTCACCTCGTTCATG	130
*GAPDH* (Control)	TCTCCTCTGACTTCAACAGCGAC	CCCTGTTGCTGTAGCCAAATTC	126

## Data Availability

The original contributions presented in this study are included in the article. Further inquiries can be directed to the corresponding author.

## Abbreviations

The following abbreviations are used in this manuscript:

FTECs	Fallopian tube epithelial cells
FDR	False detection rate
GSEA	Gene set enrichment analysis
HGSC	High-grade serous carcinoma
KEGG	Kyoto Encyclopedia of Genes and Genomes
PR	Progesterone receptor
qRT-PCR	Quantitative reverse transcription-polymerase chain reaction
FE25	P53 and Rb-defective FTECs
